# Promoted Angiogenesis and Osteogenesis by Dexamethasone-loaded Calcium Phosphate Nanoparticles/Collagen Composite Scaffolds with Microgroove Networks

**DOI:** 10.1038/s41598-018-32495-y

**Published:** 2018-09-20

**Authors:** Ying Chen, Shangwu Chen, Naoki Kawazoe, Guoping Chen

**Affiliations:** 1 Research Center for Functional Materials, National Institute for Materials Science, 1-1 Namiki, Tsukuba, Ibaraki, 305-0044 Japan; 2 Department of Materials Science and Engineering, Graduate School of Pure and Applied Sciences, University of Tsukuba, 1-1-1 Tennodai, Tsukuba, Ibaraki, 305-8577 Japan

## Abstract

Reconstruction of large bone defects remains a clinical challenge because current approaches involving surgery and bone grafting often do not yield satisfactory outcomes. For artificial bone substitutes, angiogenesis plays a pivotal role to achieve the final success of newly regenerated bone. In this study, dexamethasone-loaded biphasic calcium phosphate nanoparticles/collagen composite scaffolds with several types of concave microgrooves were prepared for simultaneous promotion of angiogenesis and osteogenesis. Microgrooves in the scaffolds were supposed to guide the assembly of human umbilical vascular endothelial cells (HUVECs) into well aligned tubular structures, thus promoting rapid angiogenesis. The scaffolds were used for co-culture of HUVECs and human bone marrow-derived mesenchymal stem cells. Subcutaneous implantation in mice showed that more blood vessels and newly formed bone were observed in the microgrooved composite scaffolds than in the control scaffold. Scaffold bearing parallel microgrooves with a concave width of 290 µm and a convex ridge width of 352 µm showed the highest promotion effect on angiogenesis and osteogenesis among the parallelly microgrooved composite scaffolds. The scaffolds bearing a grid network had further superior promotion effect to the scaffolds bearing parallel microgrooves. The results indicated that microgrooves in the composite scaffolds facilitated angiogenesis and stimulated new bone formation. The microgrooved composite scaffolds should be useful for repairing of large bone defects.

## Introduction

Reconstruction of large bone defect and non-union resulting from injuries, resections, bone tumors or failed arthroplasties has been a significant clinical challenge in orthopedics^1^. Although bone is one of the regenerative tissues, further intervention is needed when bone defect is beyond a critical size^2^. Nowadays, use of autografts and allografts remains to be the gold standard in reparation of large bone defects. However, they have some drawbacks including limited sources, risk of infection and complications, immunological rejection and donor site morbidity^3^. Consequently, more and more attention has been drawn to design artificial bone substitutes with desirable biological properties. Healing of bone defects is a complicated process resulting from the reciprocal actions of multiple cellular, molecular, biochemical and biomechanical cues, among which angiogenesis of the bone substitutes plays a pivotal role to achieve the final success of newly regenerated bone^4^. Presence of functional vascular network within the implants is essential for the timely furnishing of oxygen, nutrients and signal molecules, as well as taking metabolic waste and carbon dioxide away^5^. Blood vessel invasion happens once the engineered tissue or bone substitute is implanted. However, the vascular growth is too slow and almost negligible in providing sufficient blood supply, thus giving rise to osteonecrosis and trauma non-union, which is believed to be caused by cell death in regions far from the capillaries^6^. Due to the diffusion limit of oxygen and nutrients, generally the reconstructed tissue thicker than 400 μm should be vascularized to maintain the viability^4^. Therefore, a desirable scaffold for bone tissue engineering should have the functions to facilitate both angiogenesis and osteogenesis during bone regeneration process.

Incorporation of biological and pharmaceutical factors such as vascular endothelial growth factor (VEGF) and fibroblast growth factor in tissue engineering scaffolds has been generally used for promotion of angiogenesis^7,8^. Some concerns with this approach remain, such as instability, uncontrollable dose, high cost, short half-life and increased risk of tumorigenesis^9,10^. Therefore, development of an alternative strategy to effectively induce angiogenesis of bone substitutes and to achieve a better bone regeneration in large bone defects is strongly desirable. Recently, geometrically controlled formation of microvascular tubes of endothelial cells in microgrooved surfaces has been reported^11–13^, which is inspired by the findings that the micropatterned surfaces can orient the migration of cells^14–17^.

Along with angiogenetic function, bone tissue engineering scaffolds should also have osteoconductive and osteoinductive functions to enhance regeneration of large bone defects. Protein growth factors such as transform growth factor-β and bone morphogenetic protein-2 (BMP-2) have been frequently incorporated in scaffolds to increase their osteogenesis-promoting effects^18,19^. Nevertheless, applying protein growth factors to promote bone regeneration has the same above-mentioned problems^20^. Dexamethasone (DEX), which is an osteogenic inducer with a low molecular weight, high potency and long-acting property, has been used to replace protein growth factors for stimulation of osteogenesis^21^. DEX has been incorporated into biphasic calcium phosphate nanoparticles (BCP NPs) to combine the osteogenic effects of both DEX and the calcium and phosphorous ions released from BCP NPs^22^.

In this study, composite scaffolds with microgroove networks were prepared for simultaneous promotion of angiogenesis and osteogenesis. DEX-loaded BCP NPs (DEX-BCP NPs) were hybridized with collagen (Col) to prepare DEX-BCP-Col composite scaffolds. During preparation of the scaffolds, pre-fabricated ice particulates were used as a pore-forming agent to control pore size and increase pore interconnectivity. Furthermore, concave microgrooves were introduced in the scaffolds by using micropatterned ice lines or ice grid network. Combination of DEX, BCP NPs and collagen should have synergistic effects on osteogenic differentiation of stem cells. Microgrooves in the composite scaffolds were supposed to guide assembly of human umbilical vascular endothelial cells (HUVECs) into well aligned tubular structures, thus promoting rapid angiogenesis. The composite scaffolds were used for co-culture of HUVECs and human bone marrow-derived mesenchymal stem cells (hMSCs) to investigate their effects on angiogenesis and osteogenesis by *in vitro* cell culture and *in vivo* implantation.

## Results

### Preparation and characterization of DEX-BCP NPs and microgrooved composite scaffolds

**Figure 1:**
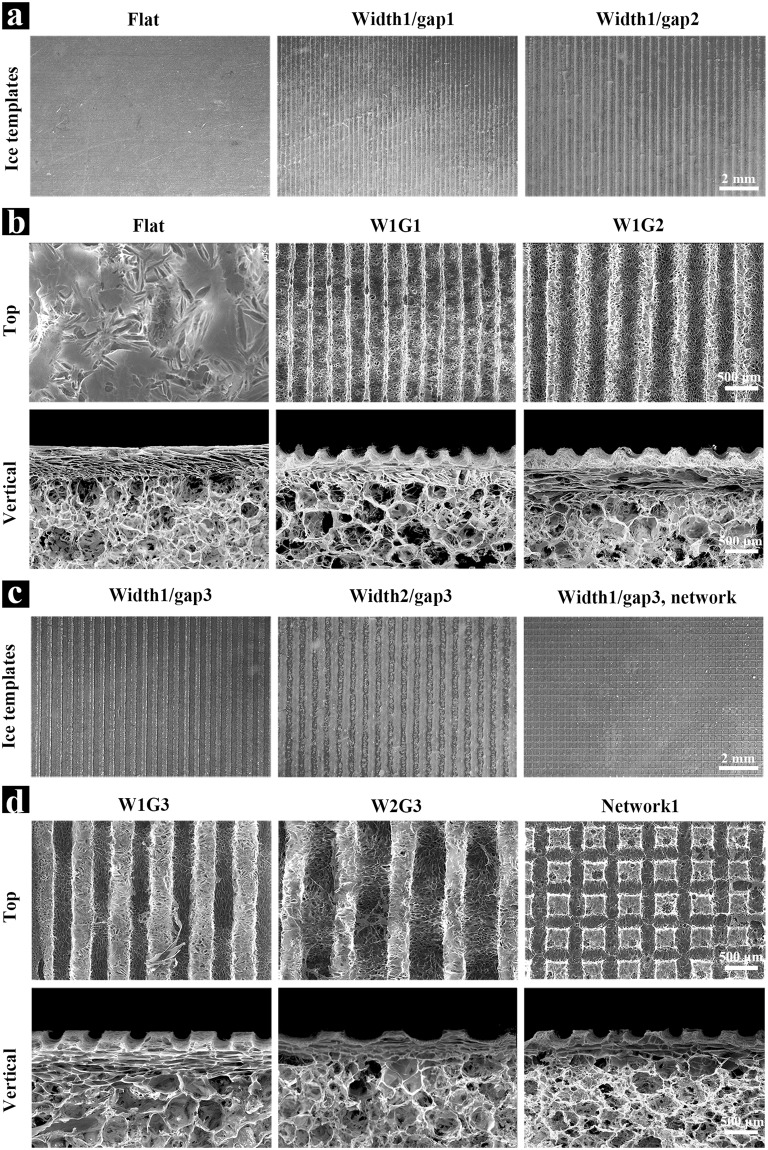
Preparation of microgrooved DEX-BCP-Col composite scaffolds. (**a** and **c**) Images of ice line templates with different dimensions and intervals prepared from water dispensing. (**b** and **d**) SEM images of microgrooved DEX-BCP-Col composite scaffolds. Flat: control DEX-BCP-Col composite scaffold without microgrooves. W1G1, W1G2 and W1G3: DEX-BCP-Col composite scaffolds bearing parallel microgrooves with a same concave width of 290 ± 21 µm and a different convex ridge width of 47 ± 8, 153 ± 15 and 352 ± 23 µm. W2G3: DEX-BCP-Col composite scaffolds bearing parallel microgrooves with a concave width of 493 ± 30 µm and a convex ridge width of 357 ± 14 µm. Network: DEX-BCP-Col composite scaffold bearing grid network of microgrooves with a concave width of 297 ± 17 µm and a convex ridge width of 346 ± 11 µm. For (**b**) and (**d**), upper images show the top view and lower images show the vertical cross-sectional view of different scaffolds.

DEX-BCP NPs were prepared by reacting DEX-contained Ca(NO_3_)_2_·4H_2_O aqueous solution with (NH_4_)_2_HPO_4_ aqueous solution. TEM observation showed the DEX-BCP NPs were short rod-like and had a dimension of 5–25 nm in width and 20–80 nm in length (Supplementary Fig. S2a). The microgrooved DEX-BCP-Col composite scaffolds were manufactured by mixing DEX-BCP NPs, collagen aqueous solution and ice particulates and placing micropatterned ice templates on top of the mixture (Supplementary Fig. S1). The constructs were freeze-dried and cross-linked to obtain the composite scaffolds. Parallel ice lines or a grid network of ice lines with different dimensions and intervals were used as templates to introduce microgrooves into the composite scaffolds (Fig. 1a,c). The flat template without ice lines or ice network was used to prepare a control composite scaffold without microgrooves. Composite scaffolds with 5 types of microgrooves were prepared by using the respective templates (Fig. 1b,d).

SEM observation showed that the basal layer of the composite scaffolds was composed of porous DEX-BCP-Col composite and the top layer was composed of microgrooved collagen thin sheet. The basal layer of each scaffold had abundant spherical large pores, most of which were interconnected by small pores (Fig. 1b,d, vertical; Supplementary Fig. S2b and c). Dimension of the spherical large pores was almost equal to the dimension of the pre-prepared ice particulates used as the pore-forming agent. The small pores provided good interconnections among the large pores. The good interconnectivity should be beneficial for migration and spatial distribution of cells. The DEX-BCP NPs were homogeneously embedded in the collagen matrices, thus making the pore surfaces rough (Supplementary Fig. S2d). The top layer of composite scaffolds had different types of microgrooves. The scaffolds having parallel microgrooves with a same concave width of 290 ± 21 µm and a different convex ridge width of 47 ± 8, 153 ± 15 or 352 ± 23 µm were designated as W1G1, W1G2 and W1G3, respectively. The scaffold having parallel microgrooves with a concave width of 493 ± 30 µm and a convex ridge width of 357 ± 14 µm was designated as W2G3. The scaffold having a grid network of microgrooves with a concave width of 297 ± 17 µm and a convex ridge width of 346 ± 11 µm was designated as Network. The microgrooves showed a semicircular shape of cross-section, which was replicated from the ice lines. The microgroove depth in Z direction was 155 ± 19 µm for W1G1, W1G2, W1G3 and Network, while 174 ± 10 µm for W2G3. The control scaffold that was designated as Flat had a relatively smooth top surface.

### Cell adhesion and distribution in the scaffolds

**Figure 2:**
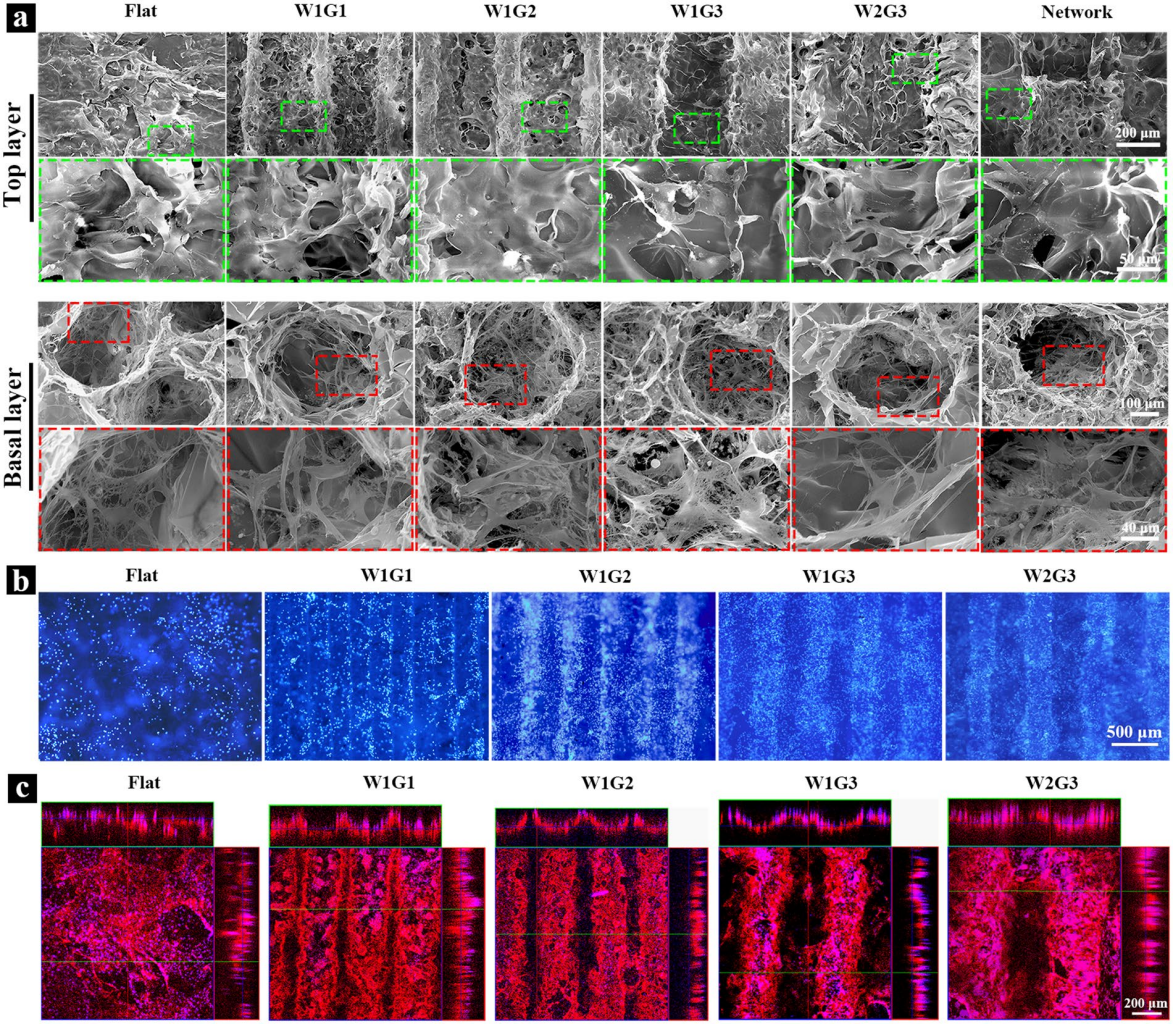
Cell adhesion and distribution in the microgrooved DEX-BCP-Col composite scaffolds. (**a**) SEM images of the scaffolds after 1 day of *in vitro* culture. Cells in the top layers and basal layers are shown at low and high magnifications. (**b**) Distribution of HUVECs after 3 days of *in vitro* culture. Blue fluorescence: cell nuclei stained by DAPI. (**c**) Assembly of HUVECs in the microgrooves after 3 days of *in vitro* culture. Red fluorescence: HUVECs immunologically stained with human-CD 31. Blue fluorescence: cell nuclei stained by DAPI.

The composite scaffolds were punched into cylindrical discs for cell culture. The hMSCs suspension solution was dropped on the basal layer side of each scaffold. After being cultured for 3 hours, the scaffold discs were turned upside down and the HUVECs suspension solution was dropped on top layer side of each scaffold. Cell adhesion in Flat, W1G1, W1G2, W1G3, W2G3 and Network composite scaffolds after 1 day culture was investigated by SEM (Fig. 2a). The hMSCs well adhered on the spherical large pores of each scaffold. HUVECs adhered on the convex ridges as well as the concave microgrooves of the microgrooved scaffold. HUVECs randomly adhered on the flat surface of the control scaffold (Flat). All cells spread widely with many filopodia. These results indicated that the scaffolds supported cell adhesion and spreading. Assembly of HUVECs on the microgrooves of the composite scaffolds was observed by DAPI staining and immunofluorescent staining of human-CD 31 after *in vitro* culture for 3 days (Fig. 2b,c). HUVECs in the Flat composite scaffold showed a random distribution. In the microgrooved composite scaffolds, HUVECs distributed predominantly in the concave microgrooves and fewer cells were observed on the convex ridges. HUVECs assembled and formed cell bands along the direction of the microgrooves.

### Gross appearance and histological analysis of *in vivo* implants

**Figure 3:**
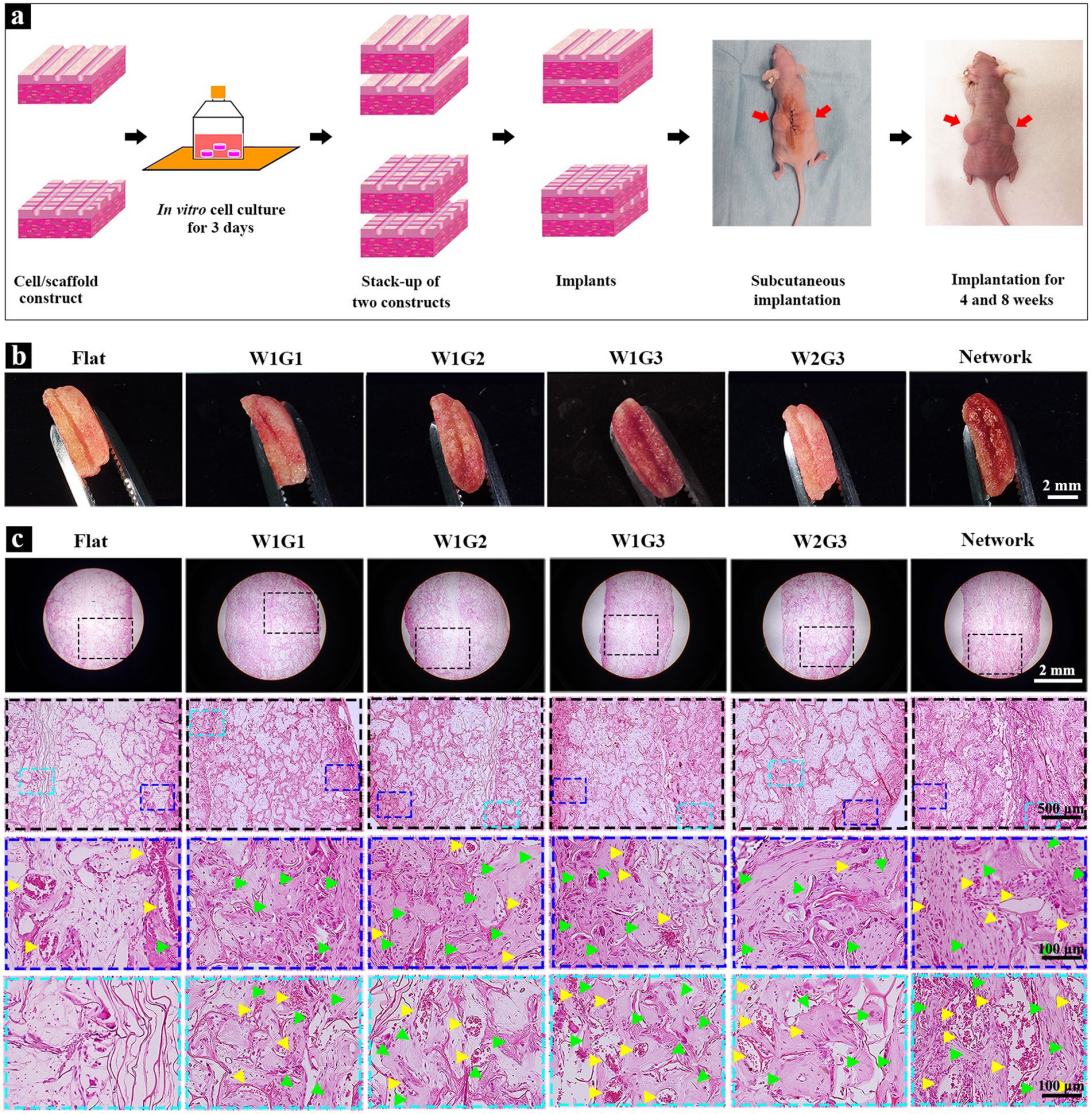
*In vivo* implantation and histological analysis of the microgrooved DEX-BCP-Col composite scaffolds. (**a**) A schematic of *in vitro* cell culture and *in vivo* implantation of the scaffolds. (**b**) Gross appearance of the implants after 8 weeks of implantation. (**c**) Photomicrographs of H&E staining of decalcified cross-sections of the implants after 8 weeks of implantation. The photomicrographs in the second line are the magnified ones of the first line. The photomicrographs in the third and fourth lines are the magnified ones at peripheral and central regions of the photomicrographs shown in the second line. Yellow triangles indicate new blood vessels. Green triangles indicate new bone formation.

To evaluate the ectopic new bone formation and angiogenesis of the scaffolds as well as to exclude influence from *in-site* bone defect microenvironment, subcutaneous implantation model was used for the *in vivo* implantation of the scaffolds. Every two constructs of cells/scaffold were stacked into one implant and subcutaneously implanted into dorsa of nude mice for 4 and 8 weeks (Fig. 3a). Gross appearance of the implants after 8 weeks of implantation is shown in Fig. 3b. The two stacked constructs of cells/scaffold were connected tightly and no obvious malposition was found, which should be due to the good clasping effect of subcutaneous model and glue effect of cell-secreted extracellular matrices. There was no evident shape change for all implants. All implants showed reddish color due to the formation of blood vessels within the scaffolds.

H&E staining showed uniform distribution of cell and extracellular matrices throughout all implants (Supplementary Figs S3 and 3c). After 4 weeks of implantation, the new blood vessels, which were recognized by the existence of lining endothelial cells and red blood cells, were observed in all groups (yellow triangles in Supplementary Fig. S3). The degree of newly formed blood vessels in peripheral regions of all implants was similar. However, in the central regions, there was different angiogenesis among the implants. The microgrooved implants had more blood vessels in the central regions than did the Flat implant. Among the 5 types of implants bearing microgrooves, angiogenesis in the central regions of W1G3 and Network appeared to be more evident than that of other groups.

**Figure 4:**
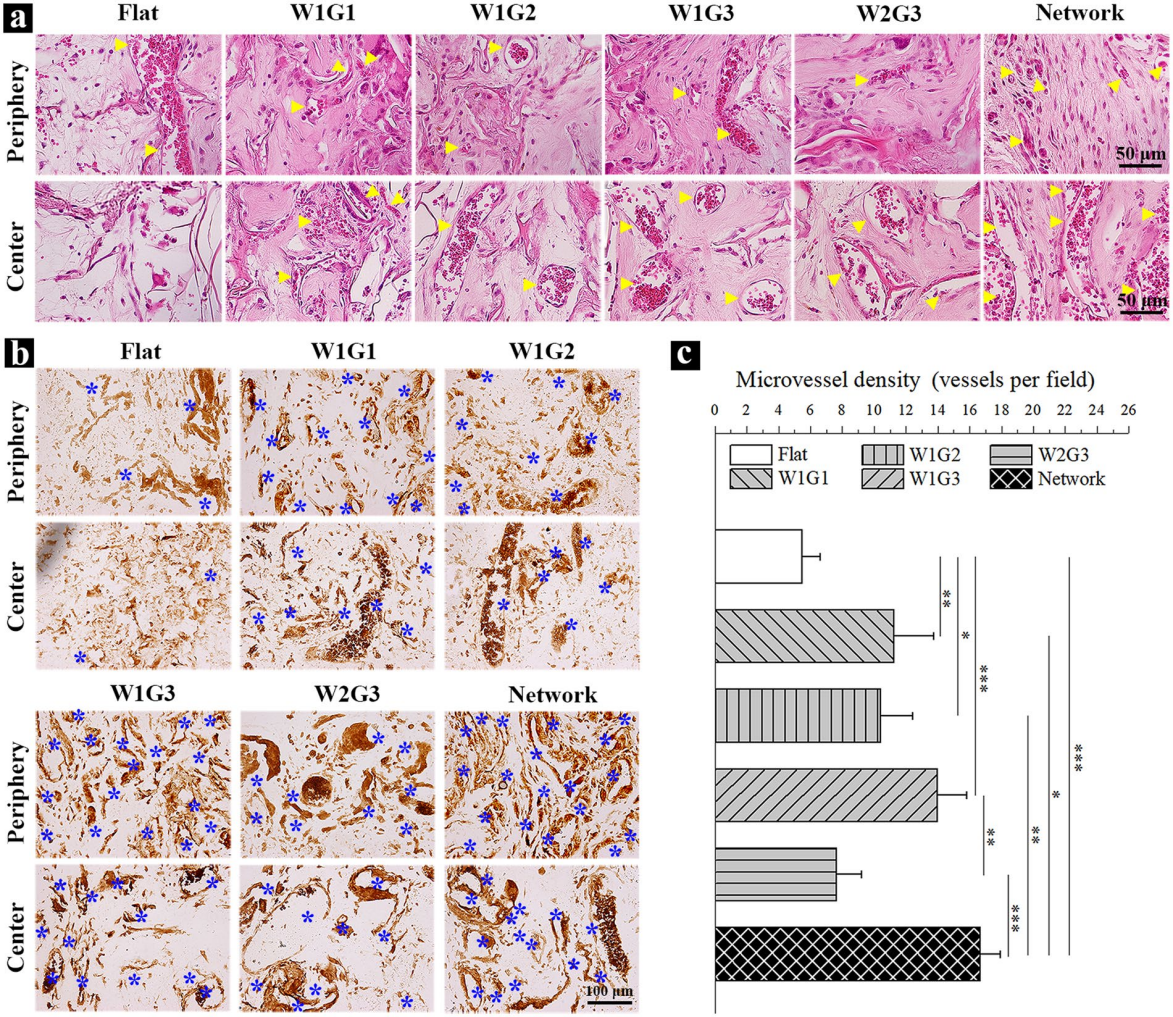
Staining and quantification of the newly formed blood vessels in the implants after 8 weeks of implantation. (**a**) Photomicrographs of H&E staining of the decalcified cross-sections of the implants at a high magnification of the peripheral and central regions. Yellow triangles indicate new formed blood vessels. (**b**) Immunohistochemical staining of vWF in the peripheral and central regions of the implants. The brown signals show the presence of vWF. (**c**) The microvessel density (MVD) within the implants. MVD was quantified by using the cross-sections immunohistochemically stained for vWF. Data represent means ± SD, n = 4. Significant difference: *p < 0.05; **p < 0.01; ***p < 0.001.

After 8 weeks of implantation, large new blood vessels were observed in all groups (yellow triangles in Fig. 3c). The new blood vessels were observed through all regions in the microgrooved implants, while they were observed only at the peripheral regions of the control Flat implant. Among the 5 types of implants bearing microgrooves, angiogenesis was more obvious in W1G3 and Network. At a high magnification, blood vessels with different sizes were observed (Fig. 4a). Some of the new blood vessels in the central regions were as large as 220 µm. Meanwhile, new bone formation was observed in all implants (green triangles in Fig. 3c). The newly formed bone was limited in peripheral regions of the Flat scaffold, while distributed in whole regions of the microgrooved composite scaffolds. In accordance with the result of angiogenesis, the degree of osteogenesis appeared higher in the Network and W1G3 scaffolds than did in other scaffolds. When the results of W1G3 and Network scaffolds were compared, angiogenesis degree and area of newly formed bone in Network scaffold were higher than those in W1G3 scaffold (Fig. 3c, Supplementary Fig. S4).

The Flat or W1G2 scaffolds without seeding cells were also stacked and implanted in nude mice for 4 weeks. H&E staining of the implants showed that some new blood vessels were observed in the peripheral regions, while no obvious angiogenesis was observed in the central regions of these two cell-free implants (Supplementary Fig. S5).

### MVD of the implants

To further visualize the newly formed blood vessels within the implants after 8 weeks of implantation, histological sections of the implants were immunohistochemically stained with von Willebrand factor (vWF), which is one kind of the endothelial cell markers (Fig. 4b). The brown-stained lumen-like structures were blood vessels. The newly formed blood vessels were observed in both peripheral regions and central regions of the microgrooved implants, while were observed only in peripheral regions of the control implant. The microvessel density (MVD) within the implants was then quantified and the results demonstrated that dimension of microgrooves influenced the density of the newly formed blood vessels (Fig. 4c). MVD of W1G1, W1G2, W1G3 and Network scaffolds was significantly higher than that of Flat scaffold. For the 5 types of microgrooved implants, the Network and W1G3 scaffolds showed higher degree of MVD than the other scaffolds, suggesting the concave width and convex ridge width of W1G3 and microgroove network were most suitable for the assembly of HUVECs to promote regeneration of blood vessels.

### Immunohistochemical staining of angiogenesis- and osteogenesis-related proteins

**Figure 5:**
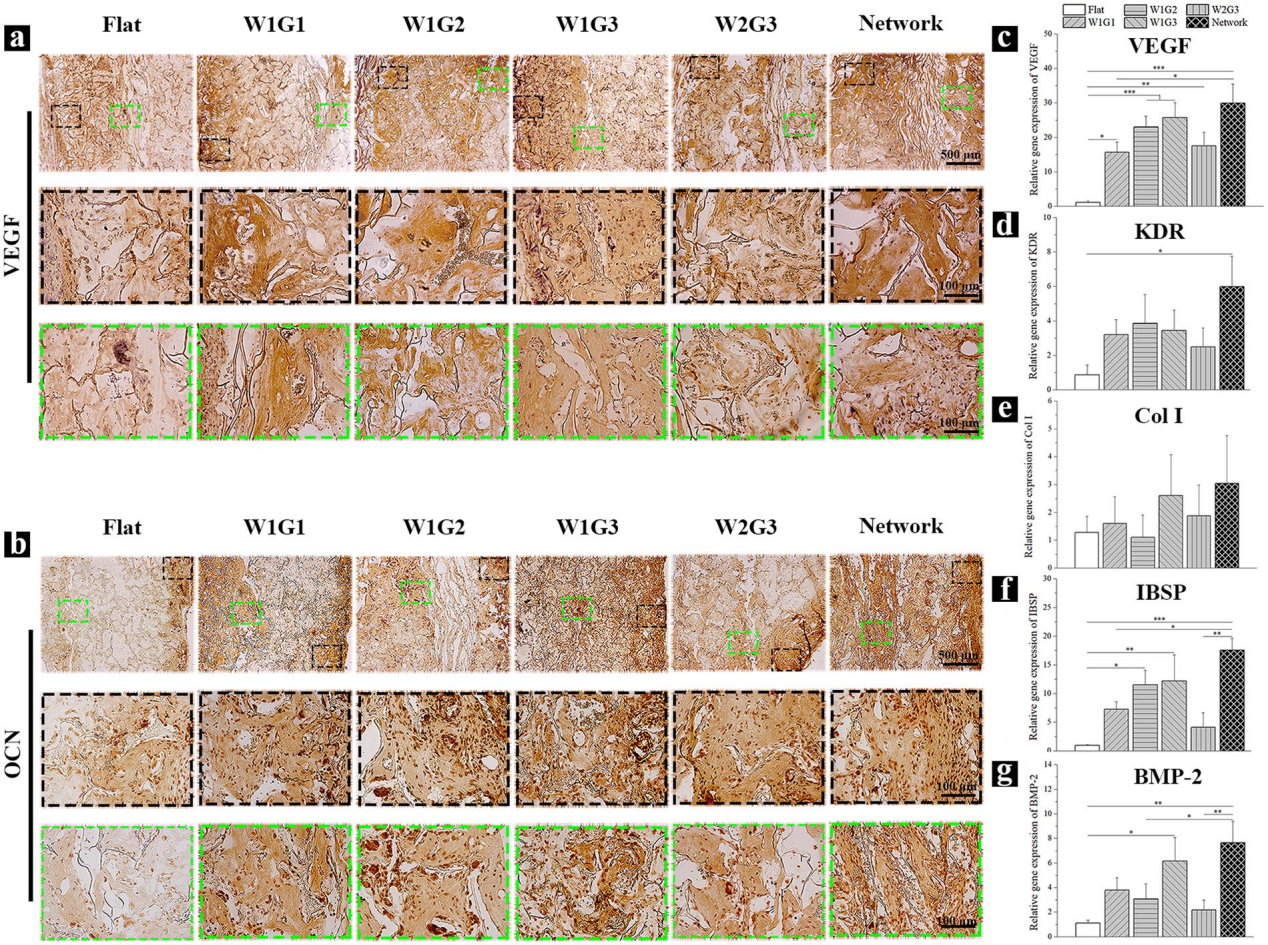
Expression of angiogenesis- and osteogenesis-related proteins and genes in the implants after 8 weeks of implantation. (**a**) Immunohistochemical staining of VEGF. The brown signals show the presence of VEGF in the cross-sections of the implants. (**b**) Immunohistochemical staining of OCN. The brown signals show the presence of OCN in the cross-sections of the implants. For (**a**, **b**), the photomicrographs in the second and third lines are the magnified ones at peripheral and central regions of the implants. (**c**–**g**) Gene expression of human VEGF (**c**), KDR (**d**), Col I (**e**), IBSP (**f**) and BMP-2 (**g**) in the implants. Data represent means ± SD, n = 4. Significant difference: *p < 0.05; **p < 0.01; ***p < 0.001.

To detect the presence of angiogenesis- and osteogenesis-related proteins in the implants after 8 weeks of implantation, immunohistochemical staining of VEGF and osteocalcin (OCN) was carried out. As a typical angiogenic growth factor, VEGF can induce the migration and proliferation of endothelial cells, leading to the formation of tubular structure^23^. VEGF protein was detected in all implants although the staining degree was different (Fig. 5a). VEGF expression in the microgrooved scaffolds was much higher than that in the Flat scaffold. VEGF showed the highest expression in Network and W1G3 scaffolds. In comparison to the Flat scaffold, VEGF showed more homogeneous distribution throughout all the regions of the microgrooved scaffolds. VEGF expression was the most homogenous in Network and W1G3 scaffolds.

OCN is secreted exclusively by osteoblasts and is involved in bone mineralization and calcium ion homeostasis^24^. OCN was expressed in all implants after 8 weeks of implantation at different degrees (Fig. 5b). OCN expression was detected only in the peripheral region of Flat scaffold, while detected throughout the whole regions of the microgrooved scaffolds. Similar to the expression of VEGF, OCN expression was higher and more uniform in the Network and W1G3 scaffolds than the other scaffolds. The results of protein expression of VEGF and OCN indicated that both angiogenesis and osteogenesis were up-regulated by the microgrooves in the composite scaffolds, among which the Network scaffold showed the highest degree of angiogenesis and osteogenesis. W1G3 scaffold showed the second highest degree of angiogenesis and osteogenesis.

### Expression of angiogenesis- and osteogenesis-related genes

Expression of angiogenic and osteogenic marker genes after *in vivo* implantation for 8 weeks was analyzed by real time polymerase chain reaction (RT-PCR) assay (Fig. 5c–g). Gene expression of VEGF was significantly up-regulated in all microgrooved scaffolds in comparison with the Flat scaffold. Gene expression of KDR (a receptor of VEGF) was significantly up-regulated by Network scaffold, while slightly up-regulated by the other microgrooved scaffolds. Gene expression of collagen type I (Col I) was slightly up-regulated by W1G1, W1G3, W2G3 and Network scaffolds. Gene expression of bone sialoprotein 2 (IBSP) was significantly up-regulated by W1G2, W1G3 and Network scaffolds, while slightly up-regulated by W1G1 and W2G3 scaffolds. Gene expression of BMP-2 was significantly up-regulated by W1G3 and Network scaffolds, while slightly up-regulated by W1G1, W1G2 and W2G3 scaffolds. For the 5 types of microgrooved scaffolds, up-regulation of angiogenic and osteogenic genes was shown at different degrees, among which the Network scaffold showed the highest degree and the W1G3 scaffold was the second highest.

## Discussion

Bone defect is one of the most common diseases that threaten public health. In recent decades, instead of allografts and autografts, more and more studies have been focused on artificial bone substitutes with suitable biological properties for repairing of large-sized bone defects. However, the current bone substitutes embrace some challenges, among which the two most crucial issues are the deficiency of mature vascular networks within the bone substitutes to transport nutrients, oxygen and signal molecules for the rapid osteogenesis^25^, and the sustained and prolonged release of valid osteogenic cues to promote and implement the long-term bone regeneration. In this study, microgrooved DEX-BCP-Col composite scaffolds were prepared to overcome these two problems. Compared to the Flat scaffold without microgrooves, the composite scaffolds bearing parallel microgrooves or microgroove network showed promotive effects on angiogenesis and osteogenesis. The microgrooves together with the released DEX, Ca^2+^ and PO_4_^3−^ were beneficial for simultaneous angiogenesis and osteogenesis. Vascular endothelial cells (VECs) are polarized cells which have an apical surface that excludes adhesion to ECM proteins and a basal surface that adheres to the underlying basement membrane proteins^13^. The cell polarity can make VECs to assemble into capillary-like networks when they are cultured on ECM such as collagen^26^. The microgrooves of composite scaffolds could induce assembly of HUVECs along the microgrooves and therefore stimulate regeneration of blood vessels. Regeneration of blood vessels could improve the supply of nutrients and removal of metabolic wastes and therefore promote migration, proliferation and osteogenic differentiation of hMSCs. Synergistic effects from the released DEX, Ca^2+^ and PO_4_^3−^ could further promote bone tissue regeneration.

To investigate the effect of microgroove structure on angiogenesis, parallel microgrooves with different concave width or convex ridge width and a grid network of microgrooves were designed and used for a comparison. Porosity of the bulk parts of all scaffolds was the same because all the scaffolds had been prepared with the same concentration of collagen and calcium phosphate and the same ratio of ice particulates^27^. At first, the effect of microgroove convex ridge width was compared by keeping the same concave width of the microgrooves. The W1G1, W1G2 and W1G3 scaffolds bearing microgrooves of the same concave width (290 µm) but different convex ridge widths (47 ± 8, 153 ± 15 and 352 ± 23 µm) were prepared and used for cell culture and implantation. HUVECs adhered well and assembled along the microgrooves in the W1G1, W1G2 and W1G3 scaffolds. The W1G3 scaffold had better promotion effect on angiogenesis than did the W1G1 and W1G2 scaffolds. A wider convex ridge width (352 ± 23 µm) was better for angiogenesis. The convex ridge width reflected the gap between microgrooves. The large gap between microgrooves (W1G3) could constrain the assembled cells in the microgrooves to facilitate formation of microtubular structure. On the other hand, the small gap (W1G1) could not protect fusion of the assembled cells in each microgroove and therefore resulted in large cell sheets.

The effect of concave width of microgrooves was compared by changing the concave width from 290 ± 21 µm (W1G3) to 493 ± 30 μm (W2G3) while keeping the convex ridge width unchanged (W1G3: 352 ± 23 µm; W2G3: 357 ± 14 µm). W2G3 scaffold showed lower promotion effect on angiogenesis than did the W1G3 scaffold. Angiogenesis in W2G3 scaffold was even lower than that of W1G1 and W1G2 scaffolds. The results indicated that the concave width of microgrooves had very evident effect on angiogenesis. The concave width of microgrooves reflected curvature of the semi-spherical microgrooves. Lower concave width resulted in larger curvature. It has been reported that curvature can affect cell-cell interaction and play an important role on tissue alignment^28^. High curvature is favorable for formation of functional multicellular structures and aligned tissues^29^. Concave microgrooves can guide the assembly of HUVECs in a contractile stress-dependent manner^30^. Initially, upon HUVECs spreading on the microgrooves, they align along the main axis of microgrooves^31^. And then, the cells spread to contact with adjacent cells, form cell-cell junctions and, due to the geometric constraints within them, exert 3D traction forces along the direction of microgrooves^31^. Cell-cell junction between endothelial cells plays a critical role in transmitting contraction stresses^32^ and generation of a mechanically balanced structure (i.e., bundles)^33^. In this study, the small concave width of microgrooves in W1G3 scaffold had high curvature, which could increase cell-cell interaction to strongly stimulate cell assembly along the long axis of microgrooves for regeneration of blood vessels. The grid network of microgrooves in Network scaffold had the same concave width and convex ridge width as those of the W1G3 scaffold. The promotion effect on angiogenesis by Network scaffold was the highest among the 5 types of microgrooved scaffolds. The superiority of Network scaffold to W1G3 and the other scaffolds should be due to the microgroove network in Network scaffold, which could provide more opportunity for HUVECs to assemble into network structure for promotion of angiogenesis.

For decades, the concept of topographic control of cell orientation has been integrated into scaffold design for tissue engineering. For instance, planar substrates with contact-printed patterns of extracellular matrices or cell adhesion proteins have been used to direct the outgrowth of neurons^34^, vascular smooth muscle cells^35^ and cardiomyocytes^36^. Microgrooved surfaces have been used to orient or align the migration of fibroblasts^15^, glial cells^14^, osteoblast-like cells^37^ and neurite outgrowths^38^. More recently, geometrically controlled tubulogenesis of endothelial cells in micropatterned surfaces has been reported^11–13^. Raghavan *et al*. have reported that when HUVECs are cultured within microscale channels coated with collagen gel, cells can organize into tubes with lumens within 24–48 hours. Moreover, the tube diameter can be controlled by varying the channel width. Cells form larger tubes as the channel width is increased from 70 and 194 to 244 µm^11^. Kenneth *et al*. have reported the formation, perfusion and maturation of 3D microvascular tubes in open cylindrical channels coated with collagen gel^12^. Chen *et al*. have reported the application of collagen porous sheets having parallel and concave microgrooves with a width of 200 μm for co-culture of HUVECs and skeletal muscle myoblasts^13^. The 3D microgrooved collagen sheets trigger spontaneous cell assembly into anisotropic bundles with well-aligned tubule-like structure^13^. All these researches reveal the function of microgrooves in guidance of endothelial cells into tubular structures *in vitro*, while our present work further confirmed the *in vivo* orientation of endothelial cells in the microgrooved scaffolds. Besides concave width, the effect of convex ridge width of microgrooves on formation of new blood vessels was also investigated in our work.

It has been commonly recognized that angiogenesis can promote osteogenesis^39,40^. The osteogenesis promotion effect of the microgrooved composite scaffolds was in a good accordance with their promotion effect on angiogenesis. The Network scaffold showed the best effect on angiogenesis and osteogenesis, followed by W1G3 scaffold. Besides the promotion effect from microgrooves, release of DEX, Ca^2+^ and PO_4_^3−^ from the composite scaffolds should also have a stimulative effect on osteogenic differentiation of hMSCs and new bone formation. Degradation of DEX-BCP NPs in the composite scaffolds resulted in a sustained release of DEX, Ca^2+^ and PO_4_^3−^, which could function synergistically to up-regulate the expression of osteogenic genes and proteins, leading to the osteogenic differentiation of hMSCs^41^. It is noticeable that the ultimately effective blood vessel formation and new bone regeneration in the scaffolds should be contributed to the close association and interaction between angiogenesis and osteogenesis. In one aspect, most osteogenic factors involved in osteogenesis, such as BMP-2, can stimulate angiogenesis either directly or indirectly by generating angiogenic molecules such as VEGF, which is involved in angiogenesis^42,43^. On the other hand, VEGF stimulates osteoblast differentiation and inhibits osteoblast apoptosis^44^. Mutual promotion of angiogenesis and osteogenesis by microgrooved DEX-BCP-Col composite scaffolds should result in the rapid formation of ectopic new bone.

Although some approaches have been explored to enhance angiogenesis and osteogenesis for bone tissue engineering, the strategy declaimed in this study exhibited some obvious advantages. Currently, most of the scaffolds designed to control cell orientation are prepared partially or completely from non-bioactive materials. Microgrooved substrates have been prepared from silicon^45^, silicone rubber^46^, titanium^47^, quartz^48^, acrylic^49^, epoxy resin^50^, polystyrene^51^, poly-lactic acid and its derivatives^52^. Whereas, in this study, the microgrooved composite scaffolds were prepared from a natural biological material, type I collagen. Collagen has many advantages such as high bioactivity, good integration of healing tissues, lowest immune and foreign-body responses, non-cytotoxicity and good biodegradation^53^. It can provide biological cues to regulate cell migration, proliferation and vascular morphogenesis of VECs^54^. Moreover, in this work, the pre-prepared ice lines were used as a template to prepare the microgrooves. This method did not require conjugation of angiogenic factors and cell adhesion ligands within the scaffolds. The microfabrication technique was a simple and mild process without use of any toxic solvents. The microgrooved DEX-BCP-Col composite scaffolds showed promotion effect on simultaneous angiogenesis and osteogenesis, which should provide a useful guide for the design of tissue engineering scaffolds.

## Conclusion

Four types of DEX-BCP-Col composite scaffolds bearing parallel microgrooves with different concave width and convex ridge width and one type of DEX-BCP-Col composite scaffold bearing microgroove grid network were prepared by using pre-fabricated ice lines as templates. The DEX-BCP-Col composite scaffolds were used for co-culture of HUVECs and hMSCs for simultaneous promotion of angiogenesis and osteogenesis. HUVECs assembled well along the microgrooves. Subcutaneous implantation in nude mice showed more angiogenesis and bone formation were observed in the microgrooved composite scaffolds than those in the control composite scaffold without microgrooves. The composite scaffolds bearing parallel microgrooves with a concave width of 290 µm and a convex ridge width of 352 µm had the highest promotion effect among the parallelly microgrooved composite scaffolds. The scaffold bearing microgroove grid network surpassed all parallelly microgrooved composite scaffolds. The results suggested that microgrooves in the composite scaffolds stimulated angiogenesis and osteogenesis. The microgrooved DEX-BCP-Col composite scaffolds provide a useful guide for large bone tissue regeneration.

## Methods

### Preparation of DEX-BCP NPs and microgrooved DEX-BCP-Col composite scaffolds

DEX-BCP NPs were synthesized by incorporation of DEX during the formation of BCP NPs^22^. Microgrooved DEX-BCP-Col composite scaffolds were prepared by generation of parallel microgrooves or microgroove grid network in DEX-BCP-Col composite scaffolds. A more detailed description is available in the experimental section and Supplementary Fig. S1.

### Characterization of NPs and scaffolds

The morphology features of DEX-BCP NPs were visualized by a transmission electron microscope (TEM, JEOL 2100F). Aqueous solution containing 10 μL of DEX-BCP NPs was dropped on a carbon-coated copper grid to prepare the samples for TEM. The top layers and cross-sections of the microgrooved DEX-BCP-Col composite scaffolds and control composite scaffold were observed by a scanning electron microscope (SEM, JSM-5610, JEOL) at an acceleration voltage of 10 kV. All SEM images were inputted to an ImageJ software (ImageJ2, NIH) to quantify the concave width, convex ridge width and depth of microgrooves in the composite scaffolds. For each type of samples, 4 images were used and every 5 microgrooves from each image were analyzed to calculate the means and standard deviations.

### *In vitro* cell culture

The composite scaffolds were punched into cylindrical discs (Φ12 mm × H1.5 mm) for cell culture. The samples were sterilized with ethylene oxide gas for 5 hours. The sterilized samples were put in 12-well cell culture plate and conditioned in Dulbecco’s Modified Eagle Medium (DMEM, Sigma-Aldrich) at 37 °C for 4 hours. The hMSCs (passage 2, Lonza) were sub-cultured using MSCBM medium (Lonza). The HUVECs (C2519A, Lonza) were sub-cultured using endothelial cell growth medium (EGM2, CC-3162, Lonza) that contained 2% FBS and VEGF. The cells were harvested using conventional trypsin/EDTA after reaching confluence and re-suspended in DMEM to prepare cell suspension solution of 2 × 10^6^ cells/mL for cell seeding. 170 μL of the hMSCs suspension solution was dropped on the basal layer side (the side without microgrooves) of the scaffold discs. After being cultured for 3 hours, the scaffold discs were turned upside down and encircled by glass rings (inner diameter: 12 mm). 2 mL of the mixture medium (1:1 MSCBM/EGM2) was added in each well and 200 μL of the HUVECs suspension solution was dropped on the top layer side (the side with microgrooves) of the scaffold discs. After culture for 6 hours in a humidified incubator at 37 °C and 5% CO_2_, the glass rings were removed and the medium was changed to the mixture medium supplemented with 10 mM β-glycerophosphate.

### Analysis of cell adhesion in the composite scaffolds

SEM was used to investigate cell adhesion and distribution in the composite scaffolds. After 1 day of culture, the cell-seeded composite scaffold discs were rinsed 3 times with PBS. The samples were then fixed with glutaraldehyde (2.5%) at RT for 1 hour. After being rinsed for 3 times with PBS and water respectively, the fixed samples were freeze-dried and their top layers and cross-sections were observed by SEM.

### Measurement of HUVECs assembly

The cells/scaffold constructs after 3 days culture were rinsed 3 times with PBS. The samples were then fixed by paraformaldehyde (4%) at RT for 15 minutes. The samples were washed with PBS, treated with Triton X-100 (0.2%) for at RT 1 hour and blocked in bovine serum albumin (BSA, 1%) at RT for 30 minutes on a rocker. Monoclonal mouse anti-human CD31 antibody (Clone JC70A, Dako) as the primary antibody was diluted in BSA (1%) at 1:40 dilution. The samples were immersed in the primary antibody-contained solution and incubated overnight at 4 °C. After being washed with PBS, the samples were then incubated with goat anti-mouse IgG secondary antibody conjugated with Alexa Fluor 594 secondary antibody (Life Technologies) at 1:500 dilution at RT for 1 hour. The cell nuclei were counterstained by DAPI solution at RT for 10 minutes. The stained samples were washed with PBS and observed by a fluorescence microscope (Olympus) or a confocal microscope (Zeiss LSM 510 Meta).

### *In vivo* implantation of the composite scaffolds

Animal experiment was approved by the Animal Experiments Committee of National Institute for Materials Science. The surgeries were carried out according to the committee guidelines. For *in vivo* implantation, the cells/scaffold constructs were prepared under the same condition as those used for *in vitro* cell culture. After 3 days of *in vitro* culture, every two constructs of cells/scaffold were stacked into one implant and subcutaneously implanted in the back of the 6-week-old athymic nude mice (Fig. 3a). 24 mice were used and each mouse was implanted with two implants. The mice were sacrificed to retrieve the implants after 4 or 8 weeks of implantation. To exclude the influence of host cells on angiogenesis and osteogenesis, the Flat or W1G2 scaffolds without seeding cells were stacked and implanted in nude mice for 4 weeks as controls. Gross appearance of the harvested implants was observed with a light microscope (DP22, Olympus Corp.).

### Histological and immunohistochemical evaluations of *in vivo* implants

The harvested implants were rinsed 3 times with PBS and fixed in neutral-buffered formalin solution (10%) at RT for 48 hours. The samples were then decalcified using decalcifying solution B (Wako Pure Chemical Industries, Ltd.) for 48 hours and dehydrated in serial dilutions of ethanol. The samples were then embedded in paraffin, cross-sectioned at thickness of 7 µm and deparaffinized. The cross-sections were stained with hematoxylin and eosin (H&E, MUTO Pure Chemicals CO., Ltd.) solution and observed under a light microscope. The cross-sections were also immunohistochemically stained for vWF, VEGF and OCN. For staining of vWF and VEGF, the deparaffinized cross-sections were performed heat-mediated antigen retrieval with citrate buffer (pH 6.0) for 15 minutes. For staining of OCN, the deparaffinized cross-sections were incubated with proteinase K for antigen retrieval for 10 minutes. And then, all the cross-sections were incubated with peroxidase blocking solution 5 minutes and goat serum solution (10%) 30 minutes. The cross-sections were respectively incubated with primary antibodies of rabbit monoclonal anti-human vWF (working concentration, 1: 100; Abcam), rabbit monoclonal anti-human VEGFA (prediluted; Abcam) and rabbit polyclonal anti-human OCN (working concentration, 5 μg/mL; Abcam) for 2 hours. After washing with PBS for three times, the cross-sections were incubated with peroxidase-labeled polymer-conjugated secondary antibodies (Cytomation EnVision+, Dako) for 1 hour, followed by incubation with 3,3′-diaminobenzidine (DAB; Liquid DAB+ Substrate Chromogen System, Dako) for 10 minutes for color development. Hematoxylin was used to counterstain the nuclei. All procedures were carried out at RT. The stained samples were observed under a light microscope.

### Evaluation of MVD

MVD has been widely performed to evaluate angiogenesis level in tumor models and pathological specimens using endothelial cell markers such as vWF or CD34^55^. Quantification of MVD was performed for the cross-sections after immunohistochemical staining of vWF. Photomicrographs taken from 5 randomly selected regions of each cross-section at a magnification of 200 were used to calculate the microvessels in each of these regions using an Image-Pro Plus software (Media Cybernetics, Inc.). The brown-stained lumen-like structures were counted as individual microvessels. The data were then processed for statistical analysis. The average value from the 5 regions was regarded as the MVD degree of each cross-section. 4 samples and every 4 cross-sections of each sample were used for the analysis.

### PCR assay for *in vivo* implants

Expression of angiogenesis-related and osteogenesis-related genes encoding human-VEGF, KDR, Col I, BMP-2 and IBSP was analyzed by RT-PCR^56^. After 8 weeks of implantation, the surrounding soft tissue of the harvested implants was cleaned. The implants were then rinsed 3 times with PBS and frozen in liquid nitrogen, followed by crushing into powder and dissolving in Sepasol solution (Nacalai Tesque, Inc.) to isolate RNA from the cells. A first stand cDNA synthesis kit (Applied Biosystems) was used to convert RNA to cDNA. RT-PCR was performed on a 7500 Real-Time PCR system (Applied Biosystems)^57^. Expression of GAPDH was used as an endogenous standard. Relative gene expression was detected by using the formula 2^−ΔΔCt^. The gene expression of cells in the DEX-BCP-Col composite scaffold without microgrooves was used as a reference. Each experiment was carried out in quadruplicate. Primers and probes are listed in Table S1.

### Statistics analysis

All quantitative data expressed as the means ± standard deviations (SD) were analyzed using a Kyplot software (version 2.0 beta 15). One-way ANOVA statistical analysis was performed to evaluate the significance of experimental data, followed by a Tukey’s post hoc test for a pairwise comparison. When *p*-value was less than 0.05, difference was considered as significant. The data are indicated by *p < 0.05, **p < 0.01 and ***p < 0.001.

## Electronic supplementary material

Supplementary material
