# Supplementary material for: Promoted Angiogenesis and Osteogenesis by Dexamethasone-loaded Calcium Phosphate Nanoparticles/Collagen Composite Scaffolds with Microgroove Networks
Source: Sci Rep. 2018 Sep 20;8:14143. doi: 10.1038/s41598-018-32495-y (PMC6147787; doi:10.1038/s41598-018-32495-y)
Supplement: Supplementary file 1 — Supplementary material [file 41598_2018_32495_MOESM1_ESM.doc]

Supplementary information

**Promoted Angiogenesis and Osteogenesis by Dexamethasone-loaded Calcium Phosphate Nanoparticles/Collagen Composite Scaffolds with Microgroove Network**

Ying Chen^1, 2^, Shangwu Chen^1, 2^, Naoki Kawazoe^1^, and Guoping Chen^*1, 2^

^1^ Research Center for Functional Materials, National Institute for Materials Science, 1-1 Namiki, Tsukuba, Ibaraki 305-0044, Japan

^2^ Department of Materials Science and Engineering, Graduate School of Pure and Applied Sciences, University of Tsukuba, 1-1-1 Tennodai, Tsukuba, Ibaraki 305-8577, Japan

* Corresponding author.

Tel: +81-29-860-4496, Fax: +81-29-860-4673, E-mail: Guoping.CHEN@nims.go.jp.

**Experimental Section**

*Preparation of DEX-BCP NPs*: DEX-BCP NPs were synthesized by incorporation of DEX during the formation of BCP NPs[^1^](#_ENREF_1). 4.5 mg of DEX (C_22_H_29_FO_5_, Sigma-Aldrich) was dissolved in 2 mL of ethanol and then added into 48 mL of 0.52 M calcium nitrate tetrahydrate (Ca(NO_3_)_2_·4H_2_O, Sigma-Aldrich) aqueous solution. The mixture solution was added dropwise to 33.4 mL of 0.5 M diammonium phosphate ((NH_4_)_2_HPO_4_, Sigma-Aldrich) solution by a syringe pump (KD Scientific Inc.). The reaction was conducted under magnetic stirring (700 rpm) in a 55 °C water bath. Throughout the reaction, ammonium hydroxide solution (NH_4_OH, Sigma-Aldrich) was added dropwise into the reaction solution to maintain the pH at 9.5. After the reaction was maintained for 30 minutes, the slurry was aged for 36 hours at room temperature (RT) to form the DEX-BCP NPs. The prepared DEX-BCP NPs were washed by dispersing them in 20 mL ethanol and shaking at 200 rpm and RT for 20 minutes followed with centrifugation at 8000 rpm. The washing with ethanol was repeated for 3 times to remove DEX adsorbed on surface of DEX-BCP NPs.

*Preparation of microgrooved DEX-BCP-Col composite scaffolds:* The microgrooved DEX-BCP-Col composite scaffolds were prepared by generation of parallel microgrooves or microgroove grid network in DEX-BCP-Col composite scaffolds which were prepared by using pre-fabricated ice particulates as a pore-forming agent (Figure S1). Pure water was spayed into liquid nitrogen to prepare the ice particulates. The ice particulates sizing between 425 and 500 μm were obtained by using two sieves sizing of 425 and 500 μm[^2^](#_ENREF_2). Porcine type I collagen (Nitta Gelatin Inc.) was dissolved in 10% (v/v) ethanol aqueous solution to prepare 2.5% and 1.0% (w/v) collagen aqueous solutions. The DEX-BCP NPs were dispersed in 10% (v/v) ethanol aqueous solution to prepare their dispersion solution at a concentration of 93 mg/mL. The ice particulates, 2.5% collagen solution and DEX-BCP NPs dispersion solution were retained in a cooling chamber (Espec Corp.) at -5 °C for 4 hours to balance their temperature to -5 °C. After temperature balance, 3 mL of the pre-cooled DEX-BCP NPs dispersion solution was added dropwise to 11 mL of the pre-cooled 2.5% collagen solution at -5 °C and mixed well. The mass ratio of NPs and collagen was 1: 1 (w/w). And then, the ice particulates were added into the collagen/NPs mixture solution at a ratio of 1: 1 (w/v) at -5 °C. The components were mixed carefully to allow the ice particulates being homogeneously distributed in the mixture solution without air bubble and then poured into silicone mold 1 (9.5 cm in length, 6.5 cm in width and 2 mm in thickness) which were placed on a copper plate wrapped by perfluoroalkoxy film (PFA film, Universal Co., Ltd.). The surface of the collagen/NPs/ice particulates mixture was platted with a spatula and a second silicone mold (9.5 cm in length, 6.5 cm in width, and 0.5 mm in thickness) was settled exactly on mold 1 and the pre-cooled 1.0 % collagen solution were filled into the second mold.

Micropatterned ice templates bearing parallel ice lines or ice grid network were prepared by a jet dispenser (MJET-3-CTR, Musashi Engineering Inc.). Dispensing nozzle of the dispenser was manipulated by a SHOT mini 200α (Musashi Engineering Inc.). PFA film-wrapped copper plate was cooled by liquid nitrogen. The jet dispenser moved back and forth upon the pre-cooled copper plate and ejected water droplets onto the plate. Water droplets were instantly frozen to form parallel ice lines or ice cross network. The size of water droplets and hence the width of each line of the micropatterns were controlled by pumping pressure in the syringe and the type of nozzle. The intervals between lines were controlled by CAD programs. 4 types of parallel ice line templates and 1 type of ice line grid network template were prepared. The micropatterned ice templates were balanced at -5 ºC for 20 minutes and then covered on the above-mentioned second silicon mold. The whole construct was then frozen at -80 °C for 8 hours and lyophilized in a freeze dryer (Wizard 2.0, SP scientific). The constructs were then cross-linked with 20 mM N-hydroxysuccimide (NHS, Wako Pure Chemical Industries, Ltd.) and 50 mM 1-ethyl-3-(3-dimethylaminopropyl) carbodiimide (EDC, Peptide Institute Inc.) in an 80% (v/v) ethanol aqueous solution at RT under gentle shaking (~ 20 rpm) for 8 hours. After cross-linking, the constructs were washed 3 times with Milli-Q water. Glycine aqueous solution (0.1 M) was used to block the unreacted NHS residues. Finally, the constructs were rinsed 6 times with water and lyophilized for usage of following experiments. 5 types of microgrooved DEX-BCP-Col composite scaffolds were prepared by using the 5 types of ice templates. They were 4 types of composite scaffolds with parallel microgrooves of different diameters and 1 type of composite scaffolds with microgroove grid network. The DEX-BCP-Col composite scaffold without microgrooves was prepared by the same procedure excepting usage of micropatterned template and used as a control.

1. Chen, Y., Li, J., Kawazoe, N. & Chen, G. Preparation of dexamethasone-loaded calcium phosphate nanoparticles for the osteogenic differentiation of human mesenchymal stem cells. *J. Mater. Chem. B* **5**, 6801-6810 (2017).

2. Zhang, Q., Lu, H., Kawazoe, N. & Chen, G. Pore size effect of collagen scaffolds on cartilage regeneration. *Acta Biomater.* **10**, 2005-2013 (2014).

**Table S1.** The primers and probes for real-time PCR.

| mRNA | Oligonucleotide |
| --- | --- |
| GAPDH | Hs99999905_m1 |
| VEGF | Hs00900054_m1 |
| KDR | Hs00911705_g1 |
| IBSP | Forward 5’-TGCCTTGAGCCTGCTTCC-3’  Reverse 5’-GCAAAATTAAAGCAGTCTTCATTTTG-3’  Probe 5’-CTCCAGGACTGCCAGAGGAAGCAATCA-3’ |
| BMP-2 | Hs00154192_m1 |
| Col I | Forward 5’-TGGCAAAGACGGTCGCAT-3’  Reverse 5’-CCTTGGGTCTGAGAGAAGTTGGT-3’  Probe 5’-GTAACCACCACCACTTG-3’ |


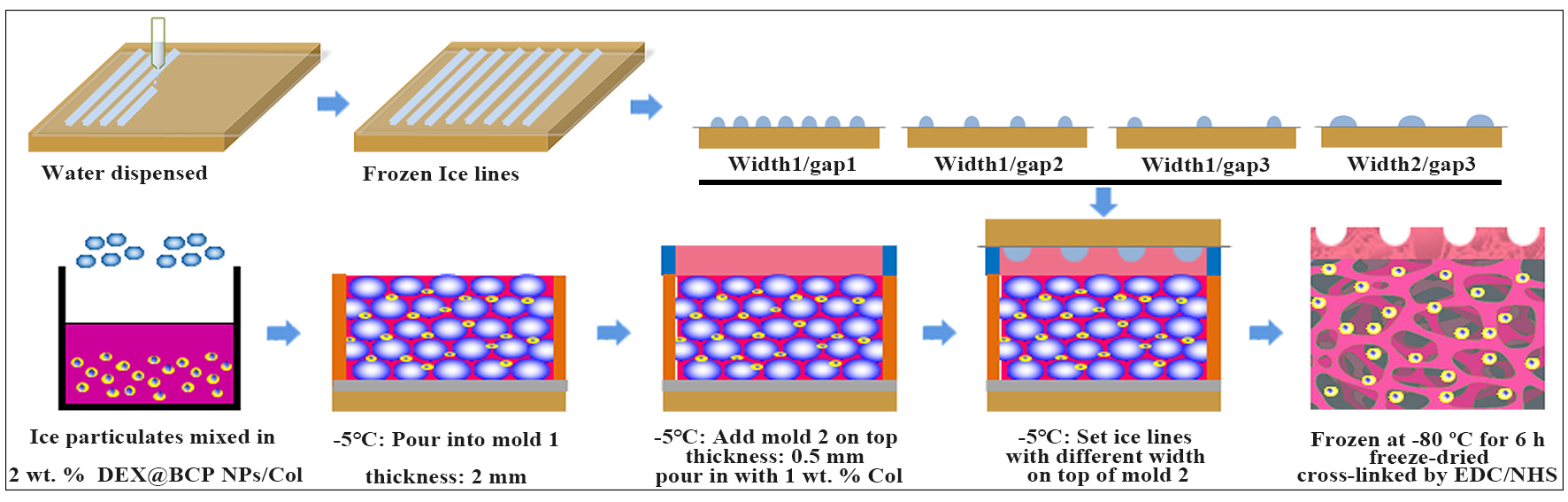


**Figure S1.** A schematic of preparation of microgrooved DEX-BCP-Col composite scaffolds.


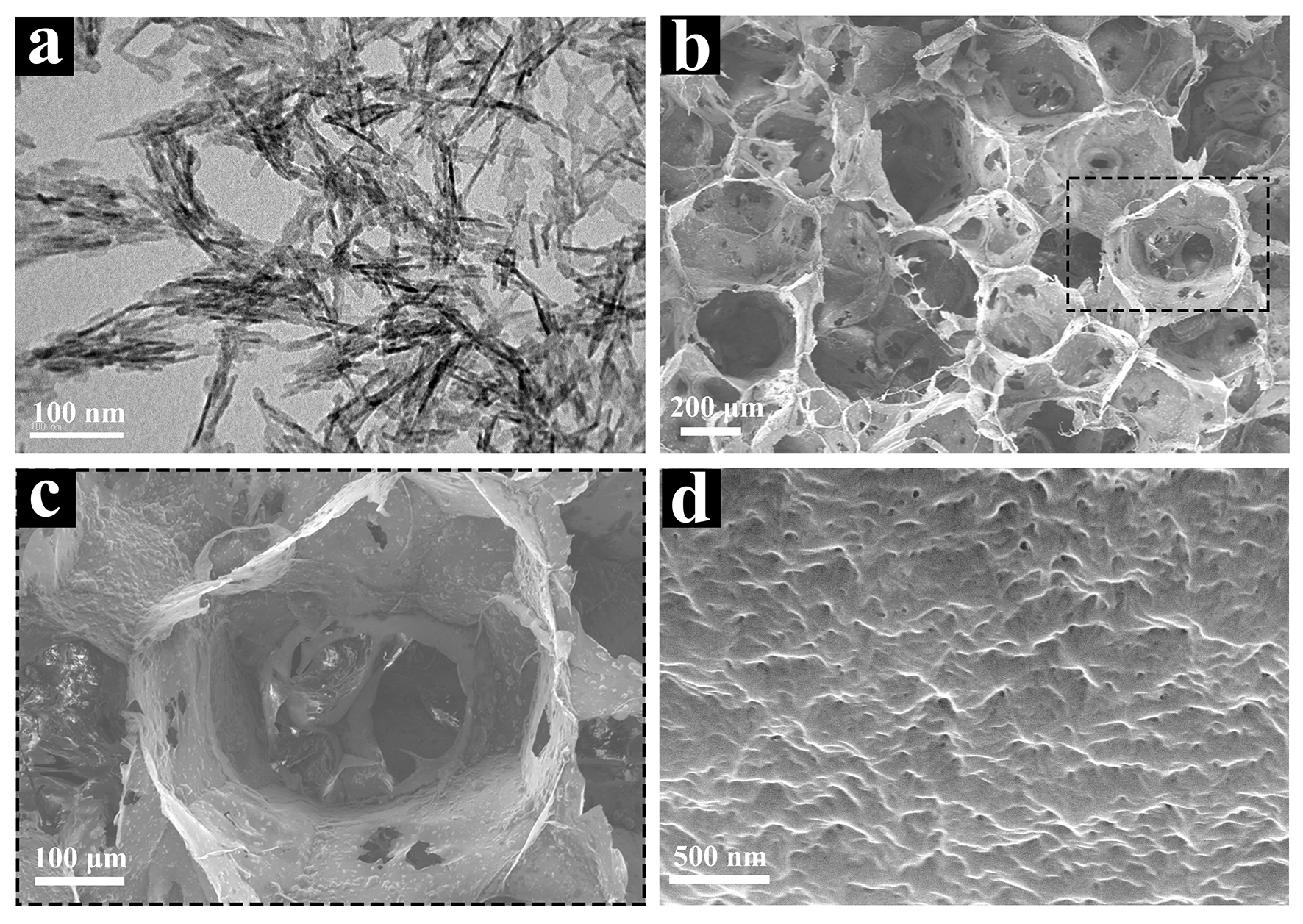


**Figure S2.** Morphology of DEX-BCP NPs and microporous structure of DEX-BCP-Col composite scaffolds. (a) Morphology of the prepared DEX-BCP NPs. (b) and (c) SEM images of the cross-sections of the basal layer of W1G3 scaffold at low and high magnifications. (d) SEM image of the pore surface of W1G3 scaffold.


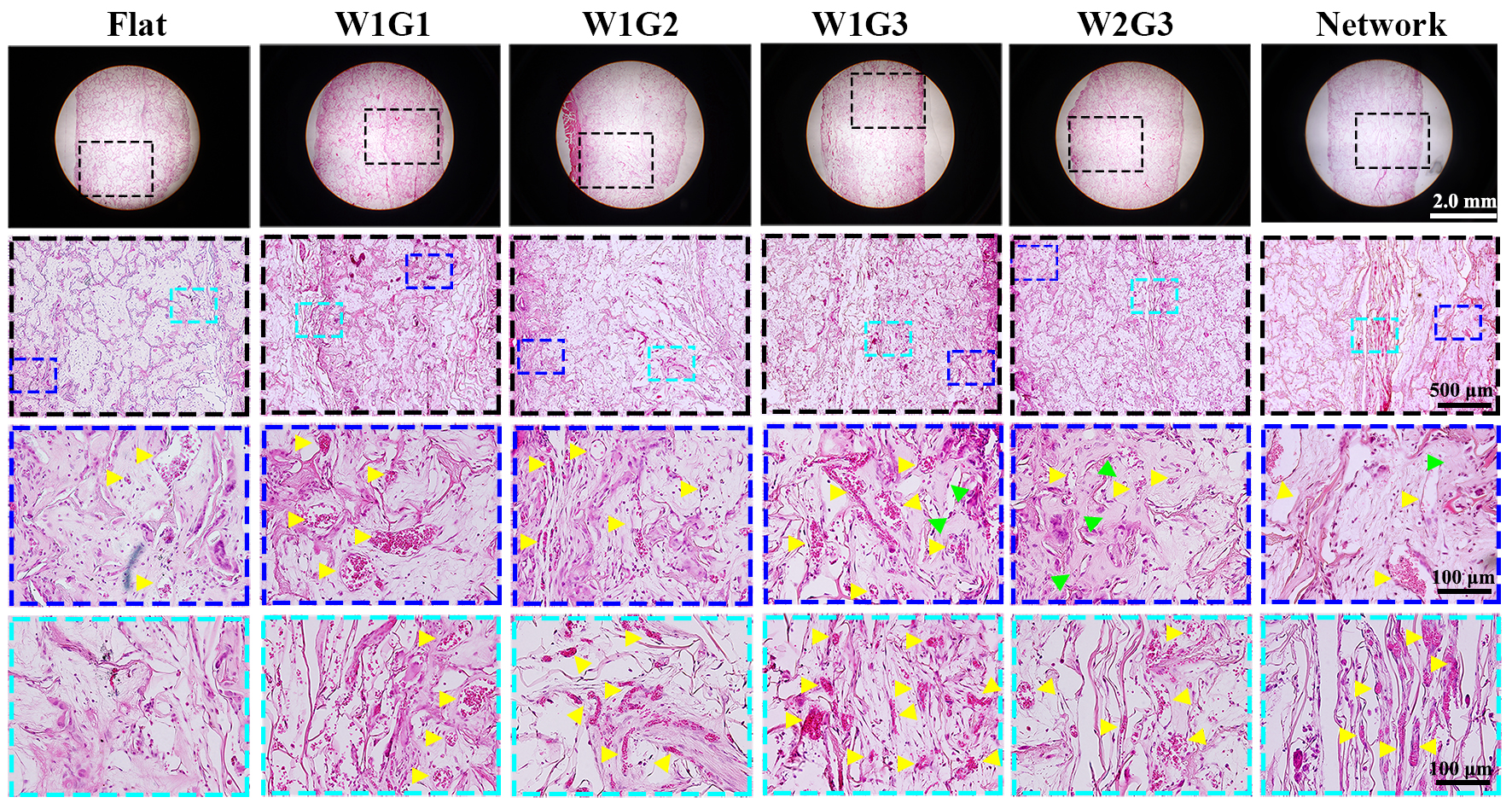


**Figure S3.** Photomicrographs of H&E staining of the decalcified cross-sections of the implants after 4 weeks of implantation. All the scaffolds were seeded with cells before implantation. The photomicrographs in the second line are the magnified ones of the first line. The photomicrographs in the third and fourth lines are the magnified ones at the peripheral and central regions of the photomicrographs shown in the second line. Yellow triangles indicate new blood vessels. Green triangles indicate new bone formation.

**
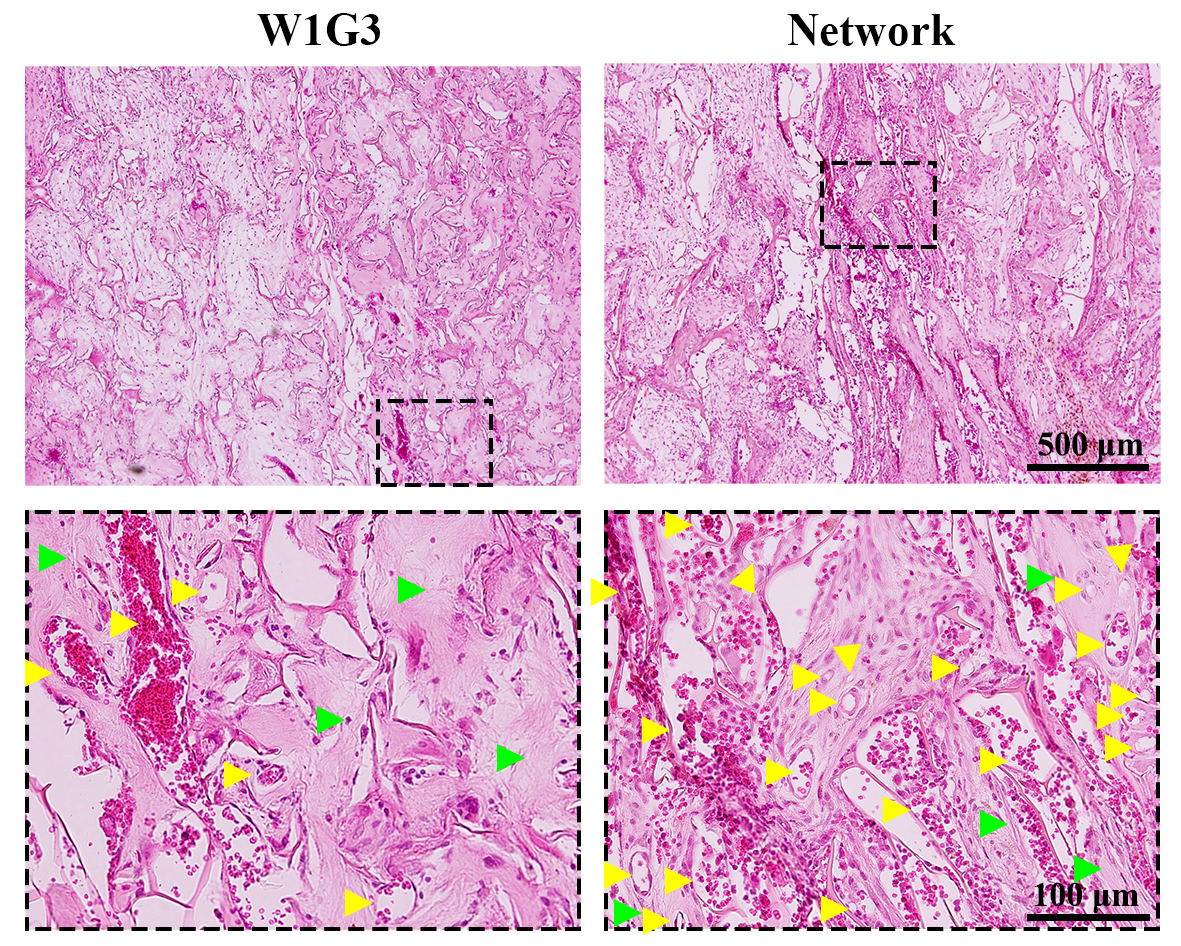
**

**Figure S4.** Photomicrographs of H&E staining of the decalcified cross-sections of the W1G3 and Network scaffolds after 8 weeks of implantation at low and high magnifications. The scaffolds were seeded with cells before implantation. Yellow triangles indicate new blood vessels. Green triangles indicate new bone formation.

**
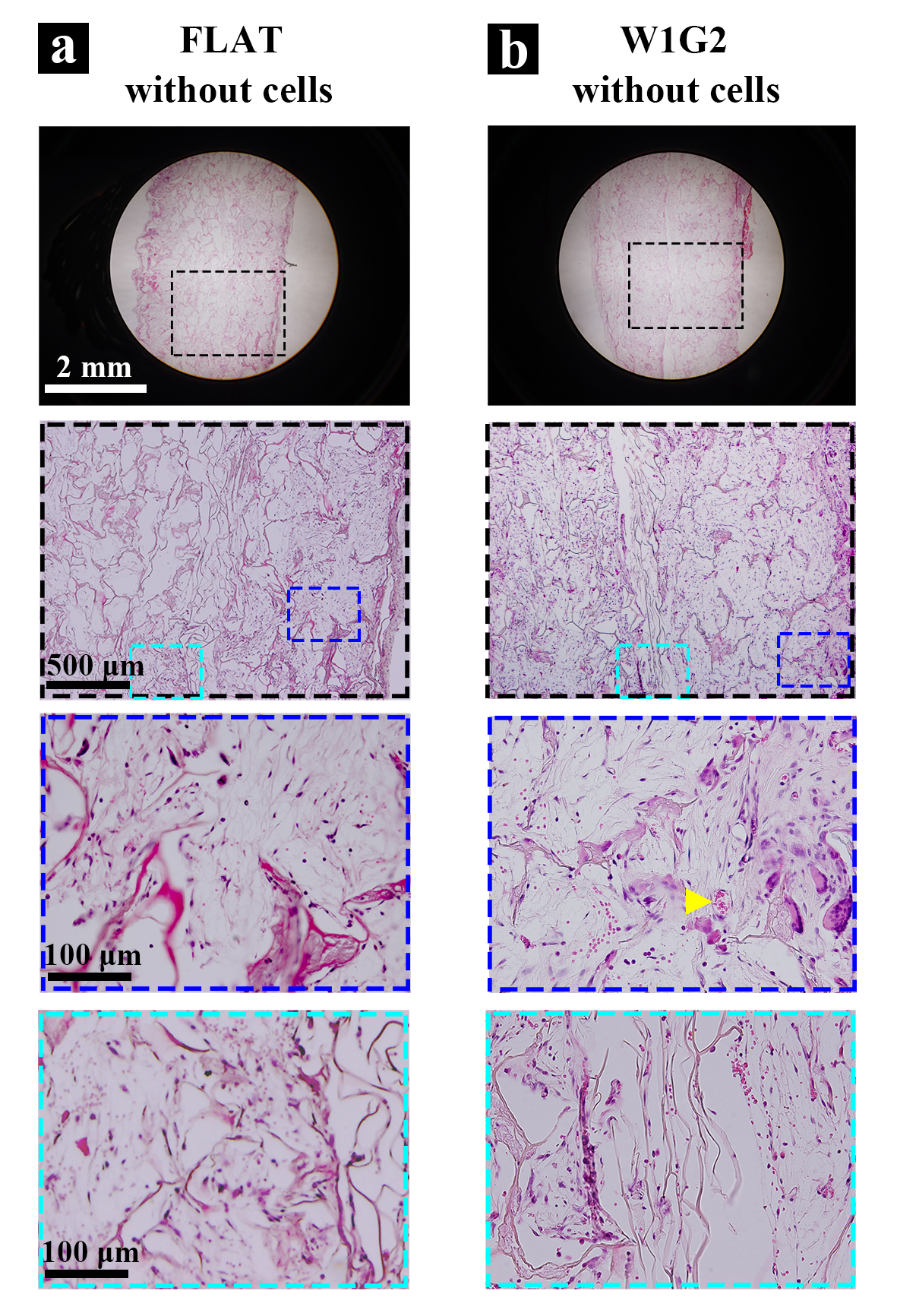
**

**Figure S5.** Photomicrographs of H&E staining of the decalcified cross-sections of the Flat or W1G2 composite scaffolds without seeding cells after *in vivo* implantation for 4 weeks. The photomicrographs in the second line are the magnified ones of the first line. The photomicrographs in the third and fourth lines are the magnified ones at peripheral and central regions of the photomicrographs shown in the second line. Yellow triangles indicate new blood vessels.
